# The cellular composition of chronic subdural hematoma

**DOI:** 10.1007/s00701-024-06101-2

**Published:** 2024-05-10

**Authors:** Thorbjørn Søren Rønn Jensen, Markus Harboe Olsen, Christina Christoffersen, Tina Binderup, Kåre Fugleholm

**Affiliations:** 1 Department of Neurosurgery, The Neuroscience Centre, Copenhagen University Hospital, Inge Lehmanns Vej 6, 2100 Copenhagen, Rigshospitalet Denmark; 2 Department of Neuroanesthesiology, The Neuroscience Centre, Copenhagen University Hospital, Copenhagen, Rigshospitalet Denmark; 3 Department of Anaesthesiology, Zealand University Hospital, Køge, Denmark; 4 Department of Clinical Biochemistry, Copenhagen, Rigshospitalet Denmark; 5 Department of Clinical Physiology and Nuclear Medicine & Cluster for Molecular Imaging, Copenhagen, Rigshospitalet Denmark

**Keywords:** Inflammatory cells, Subdural fluid, Risk profile, Cellular profile, Personalized treatment

## Abstract

**Introduction:**

The pathophysiology of chronic subdural hematoma (CSDH) remains to be fully understood. Basic knowledge of the composition and features of cells in the CSDH fluid may contribute to the understanding of the seemingly complex processes involved in CSDH formation and recurrence.

This study is the first to examine the composition of cells and of cellular features in both systemic blood and subdural fluid from CSDH patients. We hypothesized that the cellular composition and features in the hematoma fluid may be; 1) different from that in the systemic blood; 2) different between patients with and without recurrence; 3) and different between the first and second operation in patients with recurrent CSDH.

**Methods:**

Systemic blood and subdural hematoma fluid were collected from CSDH patients with and without recurrent CSDH at the time of primary and secondary surgery. Analyses of cells and cellular features included total number of white blood cells, erythroblasts, reticulocytes, platelets, neutrophilocytes, lymphocytes, monocytes, eosinophils, basophils, reticulocytes, immature granulocytes, mean corpuscular cell volume (MCV), mean corpuscular hemoglobin, mean corpuscular hemoglobin concentration, hemoglobin and hematocrit.

**Results:**

Of the 85 included patients, 20 patients were operated for a recurrent CSDH within 90 days follow-up. All cells found in the systemic blood were present in the CSDH fluid, but the composition was different (*p* < 0.0001). MCV was higher in the hematoma fluid from the primary operation of patients later developing a recurrent CSDH compared to patients not developing recurrence (*p* = 0.009). Also, the percentage distribution of inflammatory cells in hematoma fluid from patients with recurrent CSDH was different between the first and second operation (*p* = 0.0017).

**Conclusion:**

This study is the first to investigate the cellular composition of CSDH fluid. Compared to systemic blood and to a reference distribution, an increased number of immune cells were present in the hematoma fluid, supporting an inflammatory component of the CSDH pathophysiology. MCV was higher in the subdural fluid at time of the first operation of CSDH patients later developing recurrence.

**Clinical trial registration:**

The study was approved by the Scientific Ethical Committee of the Capital Region of Denmark (Journal no. H-20051073.

**Supplementary Information:**

The online version contains supplementary material available at 10.1007/s00701-024-06101-2.

## Introduction

It is well established that chronic subdural hematoma (CSDH) contains a wide range of inflammatory biomarkers, and that the outer hematoma membrane contains increased levels of white blood cells (WBC), eosinophils, neutrophils and lymphocytes [9, 16, 22, 31, 33]. The current pathophysiological understanding suggests that inflammation and neo-angiogenesis, with immature leaky blood vessels in the hematoma membrane, drives the gradual enlargement of the subdural collection, leading to delayed symptoms and diagnosis [8]. It is plausible that the original bleeding cleaves the dural border cell layer, thereby founding the subdural collection, and that well calibrated degradation and resorption of this blood, including the blood cells, may lead to complete spontaneous resolution in some patients [31, 32]. In other patients, the blood resorption is impaired leading to continuous subdural fluid accumulation from newly formed vascularized membranes. The composition of this late cellular population of the CSDH fluid may shed light on the inflammatory processes involved, and has to our knowledge not been investigated previously.

With this study we explored the composition of cells in the subdural fluid and systemic blood from patients with CSDH. We further compared cells and cellular features between 1) patients with and without recurrence, and 2) patients with recurrence between the first and second operation.

We hypothesized that the cellular composition and features in the hematoma fluid may be; 1) different from that in the systemic blood; 2) different between patients with and without recurrence; 3) and different between the first and second operation in patients with recurrent CSDH.

## Methods

### Study population

We included adult patients (≥ 18 years old) with CSDH diagnosed on computed tomography or magnetic resonance. Patients were randomly included from the Department of Neurosurgery, Copenhagen University Hospital, Rigshospitalet, Copenhagen, between January 2020 and September 2021. We excluded patients with known head trauma within 14 days of surgery and patients previously intracranial operated for other conditions than CSDH as this may cause alteration of the pathophysiology. As bilateral hematomas do not necessarily have identical cellular composition, each hematoma in patients with bilateral hematoma was considered as a separate case. Hence, bilateral CSDH was regarded as two separate cases.

Patient characteristics included known head trauma, sex, age, anticoagulant or antithrombotic treatment, performance status prior to symptom onset [29], preoperative comorbidity measured by Charlson’s comorbidity index [6], preoperative symptoms, radiological variables including midline shift, hematoma volume calculated using the XYZ/2-method [27], localization and radiological subtype [10]. Recurrent CSDH was defined as a re-accumulated, previously treated, symptomatic hematomas requiring reoperation and recorded within 90 days.

The study was approved by the Scientific Ethical Committee of the Capital Region of Denmark (Journal no. H-20051073). Consent for inclusion was obtained from either the patient or next of kin.

### Surgical approach

In Denmark CSDH surgery is standardized nationwide and only symptomatic CSDH patients are surgically treated. The surgery includes a single burrhole placed at the maximum width of the hematoma assessed by the preoperative CT-C [21]. The procedure is performed under local anesthetic, with light sedation in incorporable patients. Perioperative irrigation is followed by subdural drain placement and drainage for 24 h. Following the operation, the patients are mobilized within their limits without restriction. Postoperative CT is only indicated if symptoms lack improvement or worsen. Embolization of the middle meningeal artery or adjuvant medical treatment is not used. In case of recurrent CSDH, craniotomy can be performed on the surgeon’s decision.

### Sample collection

Hematoma fluids were collected during either craniostomy or craniotomy. In both surgical approaches, the *dura mater* was left intact following the bone opening after which the *dura mater* was opened with an intact outer hematoma membrane, and a blunt needle on a 10 ml syringe was inserted through the outer membrane. Ten milliliters of hematoma fluid were aspirated into siliconized vacuum tubes containing protamine sulfate and ethylenediamine tetraacetic acid (EDTA). If the *dura mater* was damaged and a leak of subdural fluid was observed before sample collection, the patient was excluded due to the risk of sample contamination.

Systemic venous blood was collected at time of the operation as routine blood testing.

### Cellular subtypes

To assess cell types with a possible involvement in recurrent CSDH, we measured the total number of WBC, red blood cells (RBC) and platelets both in the subdural fluid and systemic blood. WBC was measured in total and as the subtypes neutrophilocytes, lymphocytes, monocytes, eosinophils, basophils, reticulocytes and immature granulocytes (IG). RBC included erythroblasts and reticulocytes.

Furthermore, characteristics of the different cellular features were assessed by measuring of mean corpuscular cell volume (MCV), mean corpuscular hemoglobin (MCH), mean corpuscular hemoglobin concentration (MCHC), RBC deviation, hemoglobin (HGB), hematocrit (HCT), and platelet deviation also both systemically and locally in the subdural fluid.

Overall WBC count, WBC subtypes and platelets were measured as 10^9^/L. RBC was measured as 10^12^/L, MCV as 10^15^/L and MCH as 10^15^ mol. MCHC and HGB were measured as mmol/L. HCT is presented as a volume percentage of RBC in the blood.

To characterize and measure the cellular subtypes in the subdural fluid and systemic blood, a Sysmex XN-9000 analyzer (Sysmex) with extended IPU was used according to manufacturer’s instructions.

To assess the level of cells in subdural fluid in relation to the systemic blood level, a ratio between subdural fluid levels and systemic blood levels was calculated for WBC, RBC, HGB, HCT, thrombocytes, neutrophilocytes, lymphocytes, monocytes, eosinophils, basophils and reticulocytes. Furthermore, as the percentage composition of WBC subtypes may be shifted in the subdural fluid and systemic blood from patients with CSDH, we compared the percentage composition of neutrophilocytes, lymphocytes, monocytes, eosinophils, basophils and IG in the subdural fluid and systemic blood from CSDH patients to reference values considered as neutrophilocytes 65%, lymphocytes 29%, monocytes 4%, eosinophils 2%, basophils < 1% and IG < 1% [3, 13].

### Statistics

Patients’ baseline characteristics were compared between patients without recurrence and patients with recurrence. Normal distribution of data was evaluated by visual assessment of histograms. Fisher’s exact test was used for categorical variables and Student’s *t* test, or Wilcoxon rank sum test was used for continuous variables and asymmetric variables dependent on the variable distribution. Cellular profiles were compared using Wilcoxon rank sum test and presented with median difference together with 95% Hodghes-Lehmann confidence intervals. Furthermore, the prognostic value of each cell type was evaluated using receiver operating characteristics area under the curve (AUC) statistics presented with 95% confidence interval. The AUC will be interpreted as representing ‘no better than chance’ (~ 0.5), low accuracy (0.5–0.7), moderate (0.7–0.9), and high accuracy (> 0.9) [28]. The overall cellular composition was compared between the groups by Fisher’s exact test using rounded percentages of the median proportion within the group.

## Results

### Study population

We included 85 patients of which 20 had recurrent CSDH within 90 days. All patients with recurrent CSDH were male, which was different from the non-recurrence group (*p* = 0.003). Of patients with recurrent CSDH, the mean age was 74 years and the performance status before symptom onset was 0. Furthermore, we found difference in baseline demographics between patients with and without recurrent CSDH regarding hematoma localization of which 65% of patients with CSDH recurrence had bilateral hematoma compared to 31% of patients without recurrence (*p* = 0.021). Full baseline characteristics can be seen in Table 1.

**Table 1:** Patient demographics with comparison of patients with and without recurrent chronic subdural hematoma within 90 days. IQR: Interquartile range; INR: International normalized ratio; GCS: Glasgow Coma scale

	Patients without recurrence	Patients with recurrence	*p*-value
No. of patients	65	20	
Patients’ demographics			
Age, median (IQR)	75.0 (20.0)	78.0 (13.0)	0.35
Male gender, N (%)	44 (68)	20 (100)	0.003*
Performance status before symptom onset, N (%)			0.32
- 0	55 (85)	20 (100)	
- 1	3 (5)	0	
- 2	5 (7)	0	
- 3	2 (3)	0	
- 4	0	0	
**Preoperative status**			
Drugs history, N (%)			0.056
Anticoagulant treatment	14 (22)	1 (5)	
Antithrombotic treatment	12 (18)	7 (35)	
Both antithrombotic and anticoagulant treatment	0	1 (5)	
No anticoagulant or antithrombotic	39 (60)	11 (55)	
INR, median (IQR)	1.0 (0.10)	1.1 (0.12)	0.35
Thrombocytes, median (IQR)	242.5 (96)	243 (78)	0.88
Charlson’s comorbidity index, median (IQR)	4 (3)	4 (2)	0.93
Preoperative symptoms			
GCS, median (IQR)	15 (1)	15 (1)	0.99
Headache, N (%)	32 (49)	10 (50)	0.74
Vomiting, N (%)	6 (9)	2 (10)	0.92
Seizures, N (%)	3 (5)	1 (5)	0.85
Cognitive impairment, N (%)	33 (51)	14 (22)	0.13
Hemiparesis, N (%)	38 (58)	12 (60)	0.90
Aphasia, N (%)	10 (15)	4 (20)	0.60
**Radiological variables**			
Hematoma localization, N (%)			0.021*
Right	24 (37)	3 (15)	
Left	21 (32)	4 (20)	
Bilateral	20 (31)	13 (65)	
Hematoma volume, ml, median (IQR)			
Unilateral	58.5 (25.6)	68.7 (41.9)	0.88
Bilateral	119.3 (74.9)	103.9 (100.7)	1.00
Midline shift, mm, median (IQR)	7.0 (9.0)	8.0 (9.0)	0.72
Radiological subtype, N (% out of 85/33 hematomas)			0.927
Homogenous	39 (45)	17 (52)	
Separated	3 (5)	2 (6)	
Membranous	18 (21)	7 (21)	
Mixed	25 (29)	7 (21)	
Outcome			
Death, N (%)	5 (8)	3 (15)	0.328

### The cellular composition of the CSDH fluid

**Fig. 1:**
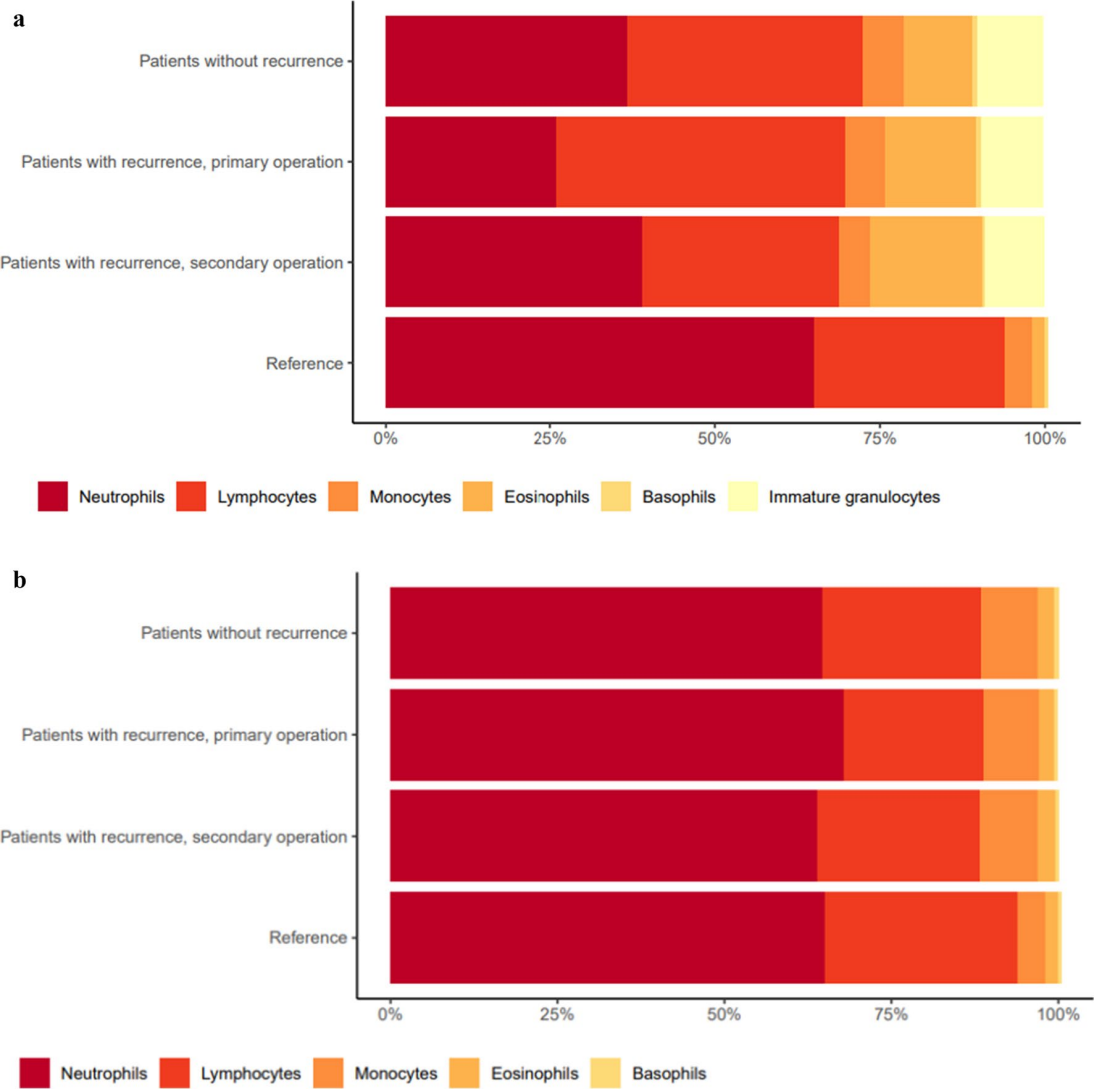
**a.** Cellular distribution in the subdural hematoma fluid. There was a significant different when comparing the cellular features in the samples from patients without recurrent CSDH (*p<*0.0001) and samples from the primary operation from patients with recurrent CSDH (*p<*0.0001) to the normal reference, and between samples from the first and second CSDH evacuation from patients with recurrent CSDH (*p*=0.0017). **b.** Cellular distribution in systemic blood samples. There was no different when comparing the cellular features between either of the groups

The composition of cells in the subdural fluid from both patients with and without recurrence was significantly different to a reference distribution (*p* < 0.0001) (Fig. 1a). A difference in the subdural cellular composition was also found between the first and second operation from patients with recurrent CSDH (*p* = 0.0017) (Fig. 1a). Especially, the presence of IG in the subdural fluid is noteworthy. The cellular composition at time of the first operation, between patients with and without recurrence, was not statistically different (Fig. 1a). The cellular composition in systemic blood showed no statistical difference when comparing either of the groups (Fig. 1b).

### Recurrence vs non-recurrence at time of first operation

**Fig. 2:**
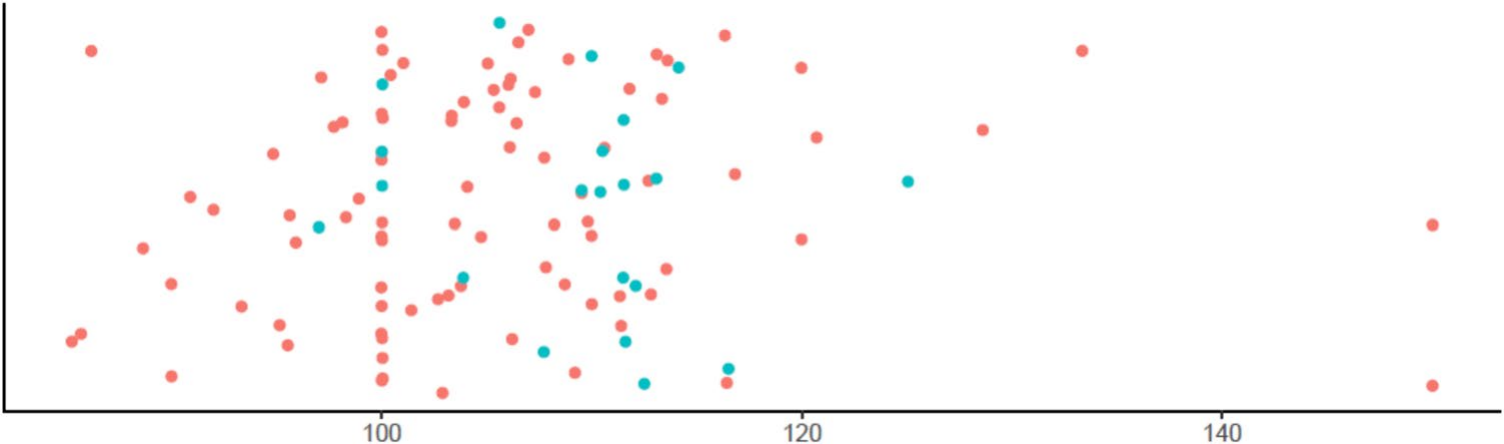
Scatter plot showing subdural levels of MCV (x-axis) at time of the first surgery stratified based on recurrence (blue dots) and non-recurrence (red dots)

When exploring a possible difference between the subdural cellular amount and the cellular features of patient with and without recurrent CSDH at the time of primary surgery, only MCV was higher in the hematoma fluid from the primary operation of patients later developing a recurrent CSDH (*p* = 0.009) (Fig. 2). We found no difference between the subtypes of granulocytes, including IG. Also, thrombocytes, lymphocytes, neutrophilocytes, reticulocytes, monocytes and the total number of WBC showed no difference.

Similar comparison was made for systemic blood, which in all the analysis was without statistical differences. Table 2 presents the comparison of cell subtypes in patients with and without recurrence at time of the first operation.

**Table 2:** Demonstration of cell type levels and cellular features in systemic blood samples, subdural fluid and the ratio between these two values (subdural/systemic) in chronic subdural hematoma (CSDH) patients with and without recurrence. P-values demonstrates the comparison between patients with and without CSDH recurrence at time of their first operation. ψHematocrit was measured as volume percentage of RBC. WBC: white blood cells; RBC: red blood cells; MCHC: *Mean corpuscular hemoglobin concentration; MCH:* Mean Corpuscular Hemoglobin; MCV: mean corpuscular volume; IQR: Interquartile range. *statistical significant difference

Cell type	Non-recurrence, N	Median 10*9/L (IQR)	Recurrence, primary operation N	Median 10*9/L (IQR)	Median difference (95% confidence interval)	p-value	AUC (95%CI)
Total number of WBC							
Subdural fluid	91	1.7 [0.94 to 4.28]	22	2.02 [0.88 to 2.64]	0.07 (-0.79 to 1.14)	0.755	0.52 (0.39 to 0.66)
Systemic blood	75	7.5 [6.6 to 9.05]	16	8.9 [6.95 to 9.9]	-1 (-2.3 to 0.4)	0.158	0.61 (0.46 to 0.77)
Ratio (subdural/systemic)	90	0.22 [0.1 to 0.5]	22	0.23 [0.12 to 0.39]	0.03 (-0.07 to 0.16)	0.462	0.55 (0.43 to 0.68)
Basophils							
Subdural fluid	75	0.02 [0 to 0.04]	18	0.02 [0.01 to 0.06]	0 (-0.01 to 0.01)	0.473	0.55 (0.42 to 0.69)
Systemic blood	73	0.03 [0.02 to 0.05]	16	0.03 [0.02 to 0.04]	0 (-0.01 to 0.02)	0.483	0.56 (0.42 to 0.69)
Ratio (subdural/systemic)	67	0.4 [0 to 1.1]	15	0.5 [0.29 to 0.79]	0 (-0.33 to 0.33)	0.832	0.52 (0.37 to 0.66)
Eosinophils							
Subdural fluid	75	0.06 [0.02 to 0.2]	18	0.18 [0.05 to 0.36]	-0.05 (-0.16 to 0)	0.074	0.64 (0.51 to 0.77)
Systemic blood	73	0.14 [0.06 to 0.26]	16	0.03 [0.02 to 0.04]	0.01 (-0.05 to 0.08)	0.72	0.53 (0.37 to 0.69)
Ratio	70	0.54 [0.14 to 1.7]	17	1.12 [0.6 to 1.75]	-0.43 (-0.91 to 0.02)	0.057	0.65 (0.53 to 0.77)
Lymphocytes							
Subdural fluid	75	0.79 [0.36 to 1.31]	18	1.08 [0.51 to 1.88]	-0.21 (-0.69 to 0.23)	0.376	0.57 (0.42 to 0.72)
Systemic blood	75	1.76 [1.22 to 2.2]	16	1.49 [1.09 to 1.97]	0.23 (-0.18 to 0.63)	0.307	0.58 (0.42 to 0.75)
Ratio	75	0.49 [0.17 to 0.74]	18	0.51 [0.38 to 0.84]	-0.03 (-0.29 to 0.15)	0.686	0.53 (0.39 to 0.67)
Monocytes							
Subdural fluid	75	0.12 [0.05 to 0.29]	18	0.13 [0.07 to 0.21]	0 (-0.06 to 0.08)	0.922	0.51 (0.37 to 0.65)
Systemic blood	75	0.63 [0.49 to 0.8]	16	0.69 [0.54 to 0.8]	-0.04 (-0.18 to 0.11)	0.643	0.54 (0.38 to 0.7)
Ratio	75	0.16 [0.07 to 0.52]	18	0.15 [0.11 to 0.29]	0.01 (-0.07 to 0.12)	0.715	0.53 (0.4 to 0.66)
Neutrophils							
Subdural fluid	75	0.56 [0.38 to 1.5]	18	0.57 [0.41 to 0.75]	0.12 (-0.11 to 0.4)	0.403	0.56 (0.43 to 0.7)
Systemic blood	75	4.8 [3.92 to 6.28]	16	5.47 [4.38 to 7.04]	-0.62 (-1.79 to 0.36)	0.203	0.6 (0.46 to 0.74)
Ratio	75	0.12 [0.06 to 0.28]	18	0.1 [0.06 to 0.14]	0.03 (-0.02 to 0.09)	0.326	0.58 (0.45 to 0.71)
Immature granulocytes							
Subdural fluid	75	0.16 [0.07 to 0.38]	18	0.16 [0.13 to 0.26]	-0.01 (-0.08 to 0.08)	0.85	0.51 (0.38 to 0.65)
Thrombocytes							
Subdural fluid	89	10 [3 to 41]	22	14 [2.75 to 22]	0 (-6 to 7)	0.841	0.51 (0.38 to 0.65)
Systemic blood	75	243 [204 to 281.5]	16	240 [209 to 294]	0 (-36 to 37)	0.975	0.5 (0.35 to 0.66)
Ratio	89	0.04 [0.01 to 0.14]	20	0.05 [0.01 to 0.12]	0 (-0.03 to 0.03)	0.944	0.51 (0.36 to 0.65)
Reticulocytes							
Subdural fluid	80	33.6 [2.6 to 95.15]	20	50.9 [11.2 to 132.75]	-13.68 (-58.8 to 8.2)	0.428	0.56 (0.4 to 0.71)
Systemic blood	70	64.5 [49 to 77.25]	15	64 [54 to 71.5]	-2 (-13 to 9)	0.699	0.53 (0.37 to 0.69)
Ratio	76	1.03 [0.11 to 3.71]	19	1.89 [0.42 to 6.45]	-0.62 (-2.09 to 0.27)	0.355	0.57 (0.41 to 0.73)
Total number of RBC							
Subdural fluid	91	1.29 [0.14 to 3]	22	2.7 [0.23 to 3.68]	-0.35 (-1.7 to 0.1)	0.224	0.58 (0.44 to 0.73)
Cellular features							
Hematocrit^ψ^							
Subdural fluid	91	0.14 [0.01 to 0.32]	22	0.29 [0.03 to 0.42]	-0.06 (-0.19 to 0.01)	0.143	0.6 (0.45 to 0.75)
Systemic blood	72	0.4 [0.37 to 0.43]	15	0.4 [0.37 to 0.44]	0 (-0.03 to 0.03)	0.866	0.51 (0.34 to 0.69)
Ratio	87	0.27 [0.03 to 0.77]	21	0.65 [0.1 to 1.03]	-0.1 (-0.44 to 0.03)	0.213	0.59 (0.44 to 0.74)
Hemoglobin (mmol/L)							
Subdural fluid	91	3.2 [0.45 to 5.55]	22	5.45 [2.02 to 7.18]	-1.5 (-3.5 to 0)	0.066	0.63 (0.48 to 0.77)
Systemic blood	75	8.2 [7.6 to 8.85]	16	8 [7.72 to 9]	0 (-0.5 to 0.8)	0.95	0.51 (0.34 to 0.67)
Ratio	90	0.41 [0.05 to 0.61]	22	0.65 [0.29 to 0.85]	-0.17 (-0.41 to 0.01)	0.074	0.62 (0.48 to 0.77)
MCHC (mmol/L)							
Systemic blood	71	20.8 [20.3 to 21.05]	15	20.6 [20.25 to 20.85]	0.1 (-0.2 to 0.5)	0.395	0.57 (0.41 to 0.73)
Subdural fluid	89	18 [16.7 to 24.5]	21	17.6 [16.7 to 18.9]	0.5 (-0.9 to 2.8)	0.447	0.55 (0.42 to 0.68)
MCV (fL)							
Systemic blood	71	93 [89.5 to 96]	15	94 [91.5 to 95]	-1 (-3 to 2)	0.476	0.56 (0.42 to 0.7)
Subdural fluid	89	103.8 [100 to 109.8]	21	110.5 [105.6 to 112.1]	-5.2 (-9.1 to -1.2)	0.009*	0.68 (0.56 to 0.8)
MCH (fmol)							
Systemic blood	70	1.9 [1.9 to 2]	15	1.9 [1.85 to 2]	0 (0 to 0.1)	0.641	0.54 (0.39 to 0.68)
Subdural fluid	88	1.94 [1.73 to 2.38]	19	1.94 [1.84 to 1.97]	0.03 (-0.09 to 0.22)	0.662	0.53 (0.41 to 0.65)

### First vs second operation in patients with recurrent CSDH

As comparison between subdural samples from the first and second operation in patients with recurrent CSDH was performed in a paired analysis, only cases with samples from both the first and second operation were included, resulting in a low number of samples. Therefore, these results must be regarded as hypothesis generating rather than definitively presenting the variation of cells and cellular features between the first and the second CSDH operation. With this in mind, we found higher levels of WBC in the subdural fluid at time of the second operation (*p* = 0.039) and higher ratio of WBC in the subdural hematoma fluid than in the blood (*p* = 0.02). Also, levels of basophils in the systemic blood were higher at time of the second operation (*p* = 0.013). Subdural levels of neutrophils were likewise higher at time of the second operation (*p* = 0.031), alongside thrombocytes which also were elevated in the systemic blood at time of the second operation (*p* = 0.031). Of the cellular features, MCHC was higher in the subdural fluid at time of the second surgery.

Full presentation of comparison of the cell subtypes and cellular features between the first and second operation in patients with recurrent CSDH can be seen in Table 3.

**Table 3:** Demonstration of levels of cell type and cellular measurements in systemic blood samples, subdural fluid and the ratio between these two values in chronic subdural hematoma patients with and without recurrence. P-values demonstrates the comparison between patients with recurrent CSDH between their first and second operation. ψHematocrit was measured as volume percentage of RBC. WBC: White Blood Cells; RBC: Red Blood Cells; MCHC*: Mean Corpuscular Hemoglobin Concentration; MCH:* Mean Corpuscular Hemoglobin; MCV: Mean Corpuscular Volume; IQR: Interquartile Range. *statistical significant difference

Cell type	Recurrence primary operation, N	Median 10*9/L (IQR)	Recurrence, secondary operation N	Median 10*9/L (IQR)	Median difference (95% confidence interval)	p-value	AUC (95%CI)
Total number of WBC							
Subdural fluid	9	0.93 [0.03 to 1.97]	9	1.63 [0.56 to 2.72]	-0.91 (-7.96 to -0.04)	0.039*	0.64 (0.37 to 0.91)
Systemic blood	18	8.45 [6.85 to 9.68]	18	8.2 [6.55 to 10.57]	0.6 (-1.35 to 1.8)	0.459	0.53 (0.33 to 0.72)
Ratio	9	0.12 [0 to 0.2]	9	0.2 [0.06 to 0.35]	-0.11 (-0.63 to -0.03)	0.02*	0.64 (0.37 to 0.91)
Basophils							
Subdural fluid	6	0 [0 to 0.05]	6	0 [0 to 0.06]	-0.06 (-0.06 to -0.06)	0.371	0.56 (0.21 to 0.9)
Systemic blood	18	0.03 [0.02 to 0.04]	18	0.03 [0.02 to 0.05]	-0.01 (-0.02 to 0)	0.013*	0.59 (0.4 to 0.78)
Ratio	5	0 [0 to 0.33]	5	0 [0 to 0.33]	-5.5 (NaN to NaN)	1	0.52 (0.17 to 0.87)
Eosinophils							
Subdural fluid	6	0.31 [0.22 to 0.62]	6	0.37 [0.08 to 1.54]	0.01 (-10.84 to 3.9)	1	0.53 (0.15 to 0.9)
Systemic blood	18	0.12 [0.08 to 0.25]	18	0.13 [0.09 to 0.23]	-0.01 (-0.13 to 0.08)	0.836	0.53 (0.33 to 0.72)
Ratio	6	2.59 [1.14 to 7.12]	6	1.16 [0.66 to 1.91]	1.43 (-32.5 to 10.14)	0.438	0.64 (0.27 to 1)
Lymphocytes							
Subdural fluid	6	0.83 [0.28 to 1.79]	6	0.97 [0.18 to 2.17]	-0.02 (-0.57 to 0.45)	0.787	0.51 (0.15 to 0.88)
Systemic blood	18	1.55 [1.21 to 1.91]	18	1.81 [1.22 to 2.45]	-0.3 (-0.89 to 0.2)	0.196	0.61 (0.42 to 0.8)
Ratio	6	0.34 [0.27 to 0.86]	6	0.36 [0.11 to 0.51]	0.26 (-0.1 to 0.74)	0.281	0.57 (0.2 to 0.93)
Monocytes							
Subdural fluid	6	0.1 [0.05 to 0.26]	6	0.17 [0.06 to 0.59]	-0.28 (-0.58 to 0.02)	0.343	0.6 (0.24 to 0.95)
Systemic blood	18	0.72 [0.54 to 0.82]	18	0.76 [0.54 to 0.8]	0.02 (-0.14 to 0.16)	0.813	0.53 (0.34 to 0.73)
Ratio	6	0.11 [0.08 to 0.38]	6	0.34 [0.12 to 0.83]	-0.3 (-0.64 to 0.05)	0.281	0.6 (0.23 to 0.96)
Neutrophils							
Subdural fluid	6	0.32 [0.2 to 0.53]	6	0.92 [0.43 to 1.26]	-0.63 (-14.87 to -0.03)	0.031*	0.75 (0.44 to 1)
Systemic blood	18	5.42 [4.34 to 6.64]	18	4.61 [3.84 to 6.41]	0.5 (-0.61 to 1.77)	0.442	0.59 (0.4 to 0.78)
Ratio	6	0.07 [0.03 to 0.11]	6	0.21 [0.13 to 0.28]	-0.15 (-3.29 to -0.01)	0.031*	0.81 (0.52 to 1)
Immature granulocytes							
Subdural fluid	6	0.14 [0.08 to 0.41]	6	0.2 [0.12 to 0.66]	-0.14 (-2.17 to 0.1)	0.219	0.57 (0.21 to 0.92)
Thrombocytes							
Subdural fluid	9	1 [0 to 15]	9	8 [3 to 13]	-2 (-9 to 50.5)	0.726	0.62 (0.34 to 0.91)
Systemic blood	17	239 [206 to 291]	17	251 [194 to 349]	-21.57 (-67.5 to -6)	0.008*	0.57 (0.37 to 0.77)
Ratio	6	0 [0 to 0.02]	6	0.02 [0.01 to 0.03]	-0.01 (-0.02 to 0.25)	0.59	0.68 (0.33 to 1)
Reticulocytes							
Subdural fluid	5	115.1 [38.1 to 115.9]	5	14.3 [10.5 to 19.6]	104.6 (-19.5 to 348.7)	0.125	0.76 (0.37 to 1)
Systemic blood	14	67 [56.5 to 72]	14	60 [51.25 to 79.75]	-1 (-20.5 to 13)	1	0.54 (0.31 to 0.76)
Ratio	3	4.42 [2.21 to 15.9]	3	0.44 [0.34 to 1.38]	8.2 (-0.24 to 25.07)	0.5	0.67 (0.01 to 1)
Total number of RBC							
Subdural fluid	9	0.36 [0.01 to 2.8]	9	0.23 [0.14 to 0.74]	0.31 (-0.14 to 1.5)	0.155	0.52 (0.2 to 0.83)
Cellular measures							
Haematocrit^ψ^							
Subdural fluid	9	0.04 [0 to 0.29]	9	0.03 [0.01 to 0.08]	0.03 (-0.01 to 0.17)	0.164	0.51 (0.2 to 0.82)
Systemic blood	17	0.4 [0.37 to 0.44]	17	0.39 [0.35 to 0.4]	0.02 (0 to 0.06)	0.058	0.63 (0.44 to 0.83)
Ratio	8	0.06 [0 to 0.45]	8	0.07 [0.03 to 0.19]	0.04 (-0.05 to 0.33)	0.313	0.56 (0.24 to 0.89)
Haemoglobin (mmol/L)							
Subdural fluid	9	2 [0 to 5.5]	9	1.2 [1.1 to 2]	1.2 (-0.7 to 3.5)	0.359	0.5 (0.18 to 0.82)
Systemic blood	18	8.15 [7.82 to 9.2]	18	7.85 [7.47 to 8.3]	0.3 (-0.15 to 0.85)	0.214	0.61 (0.41 to 0.8)
Ratio	9	0.22 [0 to 0.7]	9	0.17 [0.11 to 0.27]	0.12 (-0.08 to 0.43)	0.301	0.52 (0.2 to 0.84)
MCHC (mmol/L)							
Systemic blood	16	20.7 [20.3 to 20.92]	16	20.55 [20.25 to 21.12]	0.05 (-0.45 to 0.55)	0.9	0.5 (0.29 to 0.71)
Subdural fluid	8	18.3 [12.53 to 20.42]	8	37.35 [17.98 to 97.83]	-28.78 (-124.15 to -0.3)	0.042*	0.75 (0.5 to 1)
MCV (fL)							
Systemic blood	16	93.5 [91.75 to 95]	16	93 [89.75 to 94.25]	0.5 (-2 to 3.5)	0.676	0.53 (0.33 to 0.74)
Subdural fluid	8	107.55 [102.9 to 112]	8	106.3 [100 to 110.1]	1.61 (-5.3 to 12.9)	0.611	0.59 (0.29 to 0.88)
MCH (fmol)							
Systemic blood	16	1.9 [1.88 to 2]	16	1.9 [1.9 to 1.93]	0.05 (-0.1 to 0.15)	0.172	0.54 (0.35 to 0.74)
Subdural fluid	6	1.97 [1.86 to 2.37]	6	2.44 [2.05 to 4.29]	-0.98 (-6 to 0.33)	0.219	0.75 (0.44 to 1)

## Discussion

This study is the first to demonstrate that the subdural fluid from CSDH patients contains immune cells, RBC, and thrombocytes, and that the subdural cellular composition of granulocytes, lymphocytes and monocytes is significantly different from a reference of normal blood. A difference in the subdural cellular composition was found between the first and second operation from patients with recurrent CSDH. However, no difference of the subdural cellular composition was found at time of the primary surgery between patients with and without recurrent CSDH.

Comparing both the subdural and systemic cellular amount and the cellular features of patient with and without recurrent CSDH at the time of primary surgery, subdural MCV was significantly higher in the hematoma fluid from the primary operation of patients later developing a recurrent CSDH.

### The cellular composition in the CSDH fluid

Beside mast cells, granulocytes consist of neutrophils, eosinophils, and basophils. Lymphocytes and monocytes are WBC involved in the adaptive and innate immunity. An important finding of this study is the presentation of all the investigated immune cells and thrombocytes in the CSDH fluid. We found no literature comparing the composition of granulocytes or lymphocytes and monocytes in the subdural fluid to a reference composition of normal blood. The composition of the subdural fluid was significantly altered compared to a reference composition in peripheral blood. Interestingly, IG was present in the subdural fluid, but only constituting a small proportion of 0–0.063% in normal blood [15]. The discovery of IGs in systemic blood implies enhancement of bone marrow activity indicating an immune response. IG functions as a biomarker of inflammation in several condition including pediatric chronic kidney disease, in the prediction of perforated vs nonperforated acute appendicitis, and in the assessment of disease severity in sepsis [5, 7, 17]. Furthermore, compared to the more traditionally inflammatory markers of WBC and C-reactive protein, IG may be more effective in predicting inflammatory severity [2, 17, 23]. With the novel discovery of subdural IG in CSDH more evidence points toward pathophysiological inflammatory involvement on a cellular level.

### MCV as a risk factor for recurrent CSDH

Only MCV was significantly higher in CSDH patients later developing a recurrent CSDH compared to patients not developing recurrent CSDH. MCV is a measure of the average volume of a RBC and MCV is typically an indicator of anemia, but in cases of macrocytosis, MCV may serve as a biomarker of inflammation and endothelial function [24]. One study on tic disorder demonstrate a higher level of several hematological parameters including MCV [12]]. As RBCs act as an extracellular antioxidant system with the capability to clear exogenous reactive oxygen species and thereby, lowering inflammatory processes, the inflammatory balances may be shifted towards pro-inflammation in cases of increased RBC [12]. The indication that high MCV levels may serve as a pro-inflammatory marker is further supported by studies on schizophrenia and depression which report a higher MCV level compared to healthy controls [4, 20]. Furthermore, the inflammatory change in schizophrenia involves aberrant pro-inflammatory cytokines, which particularly are known to be higher in CSDH fluid compared to systemic blood highlighting the possible connection between MCV, cytokine levels and CSDH [11, 14, 19, 25, 26]. One mechanism connecting CSDH, inflammation and MCV is the action of pro-inflammatory cytokines, including interleukin-1ra (IL-1ra). IL-1ra is an anti-inflammatory cytokine elevated at time of the second operation in patients with CSDH recurrence [11] and IL-1ra is known to be significantly increased in the blood of patients with schizophrenia [18]. As such, an elevated level of subdural MCV can theoretically be explained in patients later developing a recurrent CSDH. However, this result should be interpretated with caution as elevated MCV in this study stand out as a single significant result in predicting CSDH recurrence. It would be adjacent to suspect a similar elevated level of other inflammatory biomarkers if the subdural inflammatory response in CSDH patients later developing recurrence truly were higher than patients not developing a recurrence.

We could not find a difference in the cellular composition either in systemic blood or subdural fluid between patients with and without recurrence at time of the first operation, which would, at least on a theoretically level, create a molecular risk profile for recurrent CSDH patients. Therefore, the cellular composition may not contribute to a specific risk profile for developing CSDH recurrence.

### Differences between cellular composition of hematoma fluid at first and second operation in recurrent CSDH

In the comparison of cellular composition between first and second operation in patients with recurrent CSDH, we found a higher level of neutrophils, MCHC and total number of WBC in the subdural fluid at time of the second operation. For all analyses the number of cases were small, and, thus, should only be considered hypothesis generating. The results from this comparison will only be discussed as supplementary (Supplementary discussion).

### Limitations

This is an explorative study with multiple comparisons of both the cell types, cellular features and composition in systemic blood and subdural fluid and there is a risk of multiple testing. This creates a possibility of statistical type I errors leading to false positive results. This could indeed be possible in the comparisons with few samples e.g., the cellular comparison between first and second operation in patients with recurrent CSDH. Therefore, these results must be regarded as exploratory only, and should be validated in a separate cohort.

Some variables in our baseline characteristics differed significantly between patients with and without recurrence. First, significantly more patients with bilateral CSDH had a recurrent CSDH. As bilateral CSDH is a known risk factor for recurrent CSDH, a skewed distribution may be expected. Male sex is a general risk factor for CSDH in the background population, but has not shown to be a risk factor for recurrent CSDH [1, 30]. Based on the differences of the baseline characteristics, the prediction of CSDH recurrence using MCV may not be applicable to female CSDH patients, though we would not expect a difference between male and female CSDH patients.

### Conclusions

This study demonstrates for the first time the cellular composition of CSDH fluid, the presence of immune cells in the CSDH fluid, and that the cell distribution in the hematoma fluid compared to systemic blood is skewed towards WBC. MCV is significantly higher in the subdural fluid at time of the first operation in CSDH patients later developing recurrence.

## Supplementary Information

Below is the link to the electronic supplementary material.

Supplementary file1 (DOCX 24 KB)

## Data Availability

The datasets generated and/or analysed during the study are available from the corresponding author on reasonable request.

## Code availability

The statistical codes used during the analyses of the study are available from the corresponding author on reasonable request.
