# Supplementary material for: The cellular composition of chronic subdural hematoma
Source: Acta Neurochir (Wien). 2024 May 10;166(1):208. doi: 10.1007/s00701-024-06101-2 (PMC11082037; doi:10.1007/s00701-024-06101-2)
Supplement: Supplementary file 1 — Supplementary file1 (DOCX 24 KB) [file 701_2024_6101_MOESM1_ESM.docx]

**Supplementary discussion**

Neutrophils are the most abundant granulocyte of WBC and one of the first responders in an inflammatory response. Thereby neutrophils are an obvious part of the inflammatory process in CSDH pathophysiology ^1^. This is supported by a study on peripheral blood in CSDH patients in which neutrophils were significantly higher compared to controls ^2^. The higher number of total WBC at time of the second operation share similar features with the higher number of neutrophils. Again, this has been proven higher in the peripheral blood of CSDH samples compared to controls ^2^.

MCHC is a ratio between hemoglobin and hematocrit, which may be linked with chronic and acute coronary syndromes due to high oxidative stress causing hemolysis leading to increased MCHC ^3^. MCHC also have predictive value for collateral carotid stenosis and may have a place as an indicator for atherosclerosis severity ^4^. This explains our finding of MCHC as a part of the pathophysiology of CSDH recurrence.

Our findings of higher level of systemic thrombocytes at time of the second surgery has also been found in similar studies on peripheral blood in CSDH patients ^5^. This was interpretated as CSDH triggering a both abnormal systematic and local inflammatory response. As the fibrinolysis-enhancing thrombomodulin is increased in CSDH fluid and the outer hematoma membrane thereby inhibiting ability of thrombin to activate thrombocytes leading to dyscoagulation, the systemic elevated level of thrombocytes may also function as a correlating attempt to locally increase the coagulation, though, this is highly speculative ^5, 6^.

Basophils were higher systemically at time of the second surgery, and, as a sub type of WBC, the higher levels can be explained by a general higher inflammatory response at time of the second surgery. This does not explain why the other systemic granulocytes were not elevated, but basophils are affected by different types of IL, which is known to be involved in CSDH development ^7, 8^.

**References for supplementary discussion**

1. Yoo SK, Starnes TW, Deng Q, Huttenlocher A. Lyn is a redox sensor that mediates leukocyte wound attraction in vivo. Nature. 2011;480(7375):109-12.

2. Fan Y, Wu D, Zhang X, et al. The inflammatory cellular feature in the peripheral blood of chronic subdural hematoma patients. J Clin Lab Anal. 2022;36(10):e24706.

3. Luke K, Purwanto B, Herawati L, Al-Farabi MJ, Oktaviono YH. Predictive Value of Hematologic Indices in the Diagnosis of Acute Coronary Syndrome. Open Access Maced J Med Sci. 2019;7(15):2428-33.

4. Urbanowicz T, Michalak M, Olasińska-Wiśniewska A, et al. Monocyte/Lymphocyte Ratio and MCHC as Predictors of Collateral Carotid Artery Disease-Preliminary Report. J Pers Med. 2021;11(12).

5. Zhang Y, Yang Y, Long S, Li G. Assessment of peripheral blood cell inflammatory markers in patients with chronic subdural hematoma. Clin Neurol Neurosurg. 2020;191:105738.

6. Murakami H, Hirose Y, Sagoh M, et al. Why do chronic subdural hematomas continue to grow slowly and not coagulate? Role of thrombomodulin in the mechanism. J Neurosurg. 2002;96(5):877-84.

7. Jensen TSR, Binderup T, Olsen MH, Kjaer A, Fugleholm K. Subdural Levels of Interleukin 1-receptor Antagonist are Elevated in Patients with Recurrent Chronic Subdural Hematomas. Inflammation. 2023.

8. Hachem CE, Marschall P, Hener P, et al. IL-3 produced by T cells is crucial for basophil extravasation in hapten-induced allergic contact dermatitis. Front Immunol. 2023;14:1151468.
